# Concurrent Exposure of Bottlenose Dolphins (*Tursiops truncatus*) to Multiple Algal Toxins in Sarasota Bay, Florida, USA

**DOI:** 10.1371/journal.pone.0017394

**Published:** 2011-03-10

**Authors:** Michael J. Twiner, Spencer Fire, Lori Schwacke, Leigh Davidson, Zhihong Wang, Steve Morton, Stephen Roth, Brian Balmer, Teresa K. Rowles, Randall S. Wells

**Affiliations:** 1 Marine Biotoxins Program, National Oceanic and Atmospheric Administration/National Ocean Service, Charleston, South Carolina, United States of America; 2 Department of Natural Sciences, University of Michigan-Dearborn, Dearborn, Michigan, United States of America; 3 Center for Human Health Risks at Hollings Marine Laboratory, National Oceanic and Atmospheric Administration/National Ocean Service, Charleston, South Carolina, United States of America; 4 Coastal Ecology Program, National Oceanic and Atmospheric Administration/National Ocean Service, Charleston, South Carolina, United States of America; 5 Chicago Zoological Society, Mote Marine Laboratory, Sarasota, Florida, United States of America; 6 Office of Protected Resources, National Oceanic and Atmospheric Administration Fisheries, Silver Spring, Maryland, United States of America; University of Glamorgan, United Kingdom

## Abstract

Sentinel species such as bottlenose dolphins (*Tursiops truncatus*) can be impacted by large-scale mortality events due to exposure to marine algal toxins. In the Sarasota Bay region (Gulf of Mexico, Florida, USA), the bottlenose dolphin population is frequently exposed to harmful algal blooms (HABs) of *Karenia brevis* and the neurotoxic brevetoxins (PbTx; BTX) produced by this dinoflagellate. Live dolphins sampled during capture-release health assessments performed in this region tested positive for two HAB toxins; brevetoxin and domoic acid (DA). Over a ten-year study period (2000–2009) we have determined that bottlenose dolphins are exposed to brevetoxin and/or DA on a nearly annual basis (i.e., DA: 2004, 2005, 2006, 2008, 2009; brevetoxin: 2000, 2004, 2005, 2008, 2009) with 36% of all animals testing positive for brevetoxin (n = 118) and 53% positive for DA (n = 83) with several individuals (14%) testing positive for both neurotoxins in at least one tissue/fluid. To date there have been no previously published reports of DA in southwestern Florida marine mammals, however the May 2008 health assessment coincided with a *Pseudo-nitzschia pseudodelicatissima* bloom that was the likely source of DA observed in seawater and live dolphin samples. Concurrently, both DA and brevetoxin were observed in common prey fish. Although no *Pseudo-nitzschia* bloom was identified the following year, DA was identified in seawater, fish, sediment, snails, and dolphins. DA concentrations in feces were positively correlated with hematologic parameters including an increase in total white blood cell (p = 0.001) and eosinophil (p<0.001) counts. Our findings demonstrate that dolphins within Sarasota Bay are commonly exposed to two algal toxins, and provide the impetus to further explore the potential long-term impacts on bottlenose dolphin health.

## Introduction

Harmful algal blooms (HABs) are commonly known for their detrimental impacts on aquatic organisms (including marine mammals), human health, and also local economies. *Karenia brevis* blooms, often referred to as “Florida red tide”, occur in the Gulf of Mexico (Florida, USA) on a nearly annual basis [1]. This dinoflagellate produces a suite of neurotoxins known as brevetoxins (PbTxs or BTXs), which are heat-stable, lipid-soluble, polyether compounds [2], [3], [4]. The proximate pharmacological target of PbTx is site 5 on the voltage-gated sodium channel [2], where they bind with high affinity (K_d_ 1–50 nM; [5]. Once bound, these toxins alter the voltage sensitivity of the channel by interfering with the voltage sensor and inactivation gate, ultimately resulting in nerve inhibition [6], [7]

Human health effects of brevetoxins generally result from neurotoxic shellfish poisoning (NSP) [8] and/or respiratory illness caused by inhalation of aerosolized toxin [9]. The former can occur following consumption of contaminated shellfish that have accumulated sufficient levels of toxin while filter feeding the algal assemblage including *K. brevis*. This illness typically affects the nervous and gastrointestinal systems; however, all symptoms are reversible and to date there have been no human deaths associated with NSP [8], [10]. Humans can also be exposed to brevetoxins in aerosolized form when the fragile, unarmored *K. brevis* cells burst due to wave action, releasing toxins into the air [11] and causing irritation and burning of the throat and upper respiratory tract [12]. In addition to brevetoxins acting as potent ichthyotoxins [13], Bossart *et al.* [14] observed brevetoxin immunoreactivity in the lung, liver, and lymphoid tissues of manatees collected during a 1996 mortality event, suggesting that brevetoxins, at least in part, can be absorbed by mammals via the inhalation of aerosolized toxins [14], [15]. Although a comprehensive understanding of brevetoxin trophic transfer is lacking, it is clear that finfish [16], [17], [18] and certain types of seagrasses (i.e., *Thalassia testudinum*) can accumulate or be associated with brevetoxins and play a primary role in brevetoxin-induced marine mammal Unusual Mortality Events (UMEs) [18], [19].

Members of the diatom genus *Pseudo-nitzschia* are associated with production of domoic acid (DA), a neurotoxin that can cause amnesic shellfish poisoning (ASP) in humans [20] and large-scale mortality of sea birds [21], pinnipeds [22], [23], and cetaceans [24]. DA is an analog of the neurotransmitter glutamate, and a partial agonist that binds with high affinity to kainate receptors and intermediate affinity to α-amino-3-hydroxyl-5-methyl-4-isoxazole-propionate (AMPA) glutamate receptor subunits [25]. Trophic transfer of DA via zooplankton into higher organisms has been well documented in krill [26], shellfish [27], sand crabs [28], and fish [23]. In the Texas region of the Gulf of Mexico, the presence of DA was first reported in phytoplankton in 1989 [29] with *Nitzschia pungens* f. *multiseries* (syn. *P. multiseries*) identified as the putative producer of DA (2.1 pg DA/cell) [29], [30]. A consortium of *Pseudo-nitzschia* species was subsequently identified (*P. pseudodelicatissima*, *P. delicatissima*, *P. multiseries*, *P. pungens*, *P. subfraudulenta*) in the nearby Louisiana coastal waters [31]. The most dominant species, *P. pseudodelicatissima*, was shown to produce DA *in situ* (up to 4.82 pg/cell) [31] and in culture (up to 36 fg DA/cell) [32]; levels that are not unlike *Pseudo-nitzschia* isolates collected from other coastal regions susceptible to DA-related UMEs and/or ASP events. A ‘hot spot’ of *Pseudo-nitzschia* spp. abundance has been identified in the northern Gulf of Mexico, near coastal Louisiana, near Mobile Bay, Alabama, and near Perdido Bay, Florida, that appears to be highly influenced by high nutrient, submarine waters [33].

As top predators, marine mammals such as bottlenose dolphins can serve as important sentinels for coastal environmental health [34], [35]. In the Gulf of Mexico, studies of the effects of *K. brevis* and brevetoxins on dolphins have historically focused on brevetoxin exposure and accumulation in carcasses recovered during UMEs [15], [18]. While data from carcasses are useful for determining relative tissue distributions and estimating amounts of toxin necessary to cause mortality, samples from live animals provide more accurate animal data (i.e., toxin levels, blood parameters, age class, sex, etc.) in conjunction with environmental (i.e., HAB species, fish collections) and spatiotemporal data. As such, animal data from live dolphins can provide insight into documenting non-lethal acute and/or chronic exposure and the effects of these exposures on a variety of other ‘health’ determinants not available from deceased animals.

Dolphin health assessments in Sarasota Bay, Florida have been ongoing since the 1980s. Because they have involved sampling members of a long-term, year-round resident community of dolphins observed since 1970 and spanning at least five generations, these health assessments have provided a unique opportunity to monitor long-term trends of a dolphin population [36]. Samples obtained between 2003 and 2005 have shown consistent exposure to brevetoxin when *K. brevis* was present in the surrounding water [37]. The major route of exposure of these animals to brevetoxin appeared to be via consumption of finfish such as pinfish (*Lagodon rhomboides*), pigfish (*Orthopristis chrysoptera*), striped mullet (*Mugil cephalus*), and spot (*Leiostomus xanthurus*) [17], which are among the primary prey items for bottlenose dolphins in the Sarasota Bay region [38], [39]. Although *K. brevis* blooms have been shown to affect fish population diversity and community structure [40], detectable levels of brevetoxin have been identified in shellfish and finfish for up to one year following a *K. brevis* bloom [16], [17], [41]. In contrast to studies involving brevetoxin, there have been no studies examining the presence or effects of DA or DA-producing *Pseudo-nitzschia* species on the dolphin population in Sarasota Bay.

The primary objective of this study was to assess the exposure of live dolphins from the Sarasota Bay area to both brevetoxin and DA. Dolphin tissue/fluid and environmental samples were obtained from an intensive sampling effort in May 2008 and archived dolphin samples were retrospectively assessed back to June 2000. These data have been used to determine the extent of exposure of a marine mammal species to multiple algal toxins and assess putative health effects that may have negative impacts on this population.

## Methods

### Ethical treatment of animals

This study was carried out in strict accordance with the U.S. Marine Mammal Protection Act. Protocols for the dolphin capture-release and tagging were conducted under National Marine Fisheries Service Scientific Research Permits No. 522-1569 and No. 522-1785 issued to RSW. Research conducted under these permits was approved through the Mote Marine Laboratory annual Institutional Animal Care and Use Committee (IACUC) reviews.

### Phytoplankton cell abundance

**Figure 1:**
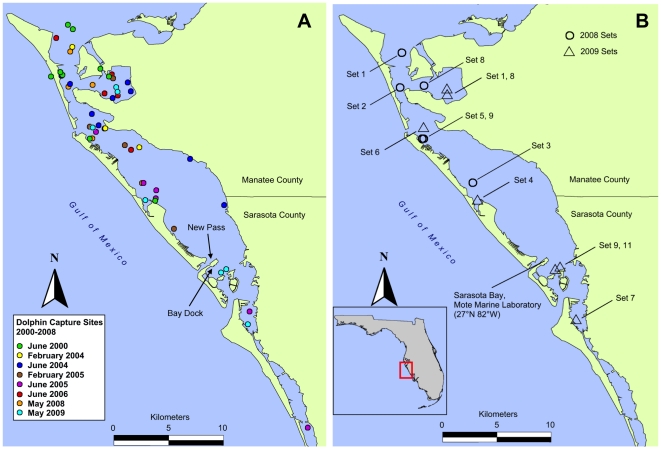
Locations of live captured bottlenose dolphins (*Tursiops truncatus*) in Sarasota Bay, Florida, USA. Shown are (A) the captured locations for all sampled animals between 2000 and 2009 and (B) the specific 2008 and 2009 set sites where additional water sampling was also performed.

Multiple-year *Karenia brevis* cell counts were determined on a nearly daily basis at two designated sampling sites in the waters adjacent to Mote Marine Laboratory (Bay Dock: lat./long. 27.33253/-82.57783 and New Pass: lat./long. 27.33382/-82.57911) (Figure 1) and average cell counts served as proxy indicators of HAB activity within the bay. Additional water samples were collected for cell counts adjacent to various dolphin capture-release sites in 2004 (February), 2008, and 2009. For all samples, the direct microscopic cell enumeration method used had a detection limit of 1000 cells/L. Prior to the May 2008 dolphin health assessment, much of the data for *Pseudo-nitzschia* spp. cell abundance were not quantified but limited to presence or absence only. During the May 2008 and 2009 dolphin health assessments phytoplankton were collected from surface waters either directly into sample collection tubes or using a plankton net (model 9100-10, Sea-Gear Corporation, Melbourne, FL, USA) with a 10 µm mesh size. All plankton samples were preserved with ∼1% acidified Lugol's iodine solution. Concentrated net tow samples and whole water samples concentrated using Utermöhl's sedimentation chambers [42] were microscopically observed using an Olympus BX-51 (Center Valley, PA, USA) microscope with differential interference contrast (DIC). Samples with potential HAB species were positively confirmed using scanning electron microscopy (SEM) where samples were prepared using Simonsen's method for cleaning diatom frustules [43]. SEM samples were prepared following the method outlined in Morton [44] and examined with a JEOL 5600LV SEM (Tokyo, Japan) operated at 15 kV.

### Benthic sediment and snail collections

To assess possible benthic routes of toxin trophic transfer, snails and sediment samples were collected from several health assessment sites using a 25 µm Fisher Scientific standard test sieve. Approximately 500 g of the upper 5 cm of the sediment layer was collected and drained of seawater, and whole snails (0.7–1.6 mm shell length) contained in this sediment layer were collected and stored frozen (−20°C) in 50 mL polypropylene centrifuge tubes. Fifty grams of the remaining sediment was collected and stored in similar manner. Sediment samples were subsequently centrifuged at 2500× *g* and again drained of seawater prior to toxin extraction.

### Fish collections

In 2008, various fish species were collected by cast net in the study area on 5/1/2008 (set site 1, lat./long. 27.50841/-82.69359) and 5/7/2008 (Chicago Zoological Society; lat./long. 27.33160/-82.57790) (see Figure 1B). In 2009, multiple fish species were collected by cast net in the study area on 5/5/2009 (*ca.* set site 4, lat./long. 27.395157/-82.629472) and 5/7/2009 (*ca.* set sites 1 and 8, lat./long. 27.47256/-82.661086) (see Figure 1B). Whole fish were identified, sorted according to species, weighed, and frozen at −20°C. Whole viscera (all organs in intracoelomic cavity) were dissected, weighed, and extracted for biotoxins.

### Dolphin samples

Dolphin samples (urine, feces, whole blood, serum, and gastric fluid) for this study were collected from individuals sampled during health assessments in June 2000, February 2004, June 2004, February 2005, June 2005, June 2006, May 2008, and May 2009 (Figure 1A) according the methods of Wells *et al*. [34]. Briefly, dolphins were captured by encircling them with a 500 m×4 m seine net deployed by a local commercial fisherman from a net boat in shallow (<2 m) waters. May 2008 and 2009 capture and release sites (“set sites”) are illustrated in Figure 1B. A team of more than 50 biologists, veterinarians, and trained dolphin handlers operating from up to eight other small boats was involved to insure the safe handling of the animals. Once secured, the dolphins were transferred one by one onto the padded, shaded deck of a 9 m veterinary examination boat where a series of length and girth measurements were taken, as well as weight. Whole blood (∼10 mL aliquot) was collected via venipuncture of the fluke vasculature and was (1) separated by centrifugation into plasma and serum for subsequent hematologic and biochemical analysis at a diagnostic laboratory as previously described [34], [45], [46], and (2) spotted onto blood collection cards for toxin analysis as described by Maucher et al. [47]. Urine samples were collected via the urethra using a sterile catheter. Gastric samples were collected using a small tube inserted via the esophagus into the stomach, from which stomach contents were drained and stored. Feces samples were taken during processing using a sterile catheter. All samples were collected into sterile cryotubes or centrifuge tubes and frozen until biotoxin analysis was conducted. Due to the retrospective nature of this study, many pre-2008 samples were initially stored at −20°C prior to permanent storage at −80°C. Specific site and animal information for 2008 and 2009 are outlined in Table 1.

**Table 1:** Animal information and biotoxin tissue/fluid concentrations for the bottlenose dolphins (*Tursiops truncatus*) sampled during the May 2008 and 2009 health assessments in Sarasota Bay, Florida.

Animal ID (FB#)	Capture Date	Set #	Sex	Age	Length (cm)	Weight (kg)	Brevetoxin Concentration (ng/g or ng/mL)						Domoic Acid Concentration (ng/g or ng/mL)					
							Blood	Serum	Urine	Fecal	Gastric Fluid	Milk	Blood	Serum	Urine	Fecal	Gastric Fluid	Milk
133	5/1/2008	1	F	9	225	148	<dl	<dl	<dl		5.2		<dl	<dl	<dl		<dl	
224	5/1/2008	1	M	6	213	120	<dl	<dl	<dl	13.6	<dl		<dl	<dl	17.6	41.5	<dl	
203	5/2/2008	2	F	8	217	135	<dl	<dl	<dl		5.6		<dl	<dl	1.8		<dl	
155	5/5/2008	3	F	18	240	171	<dl	<dl	<dl	30.0	6.5		<dl	<dl	1.6	9.4	<dl	
205	5/5/2008	3	F	2	183	72	<dl	<dl	<dl		4.1		<dl	<dl	<dl		<dl	
197	5/5/2008	3	F	5	210	118	<dl	<dl	<dl		3.7		<dl	<dl	<dl		<dl	
175	5/6/2008	5	F	17	na	na	<dl	<dl					<dl	<dl				
207	5/6/2008	5	F	3	190	98	<dl	<dl	<dl		5.4		<dl	<dl	4.9		13.8	
209	5/8/2008	8	F	4	198	92	<dl	<dl	<dl		4.5		<dl	<dl	6.1		<dl	
11	5/9/2008	9	F	24	257	204	<dl	<dl	<dl	13.6	6.7		<dl	<dl	1.0	10.3	<dl	
250	5/9/2008	9	M	3	190	89	<dl	<dl	<dl	3.5	10.5		<dl	<dl	4.4	8.5	<dl	
178	5/9/2008	9	M	13	257	201	<dl	<dl	<dl	12.3	4.6		<dl	<dl	<dl	<dl	<dl	
188	5/9/2008	9	M	12	236	173	<dl	<dl	<dl	2.3	4.3		<dl	<dl	<dl	<dl	<dl	
151	5/3/2009	1	F	9	228	130	<dl	<dl	<dl	<dl	<dl		<dl	<dl	1.6	37	<dl	
55	5/4/2009	4	F	23	250	176	<dl	<dl	<dl	3	<dl	<dl	<dl	<dl	trace	4	<dl	<dl
213	5/4/2009	4	F	2	187	71	<dl	<dl	<dl		<dl		<dl	<dl	<dl		<dl	
138	5/4/2009	4	M	17	265	240	<dl	<dl	<dl	3	<dl		<dl	<dl	3.6	11.5	<dl	
92	5/5/2009	6	M	21	249	193	<dl	<dl	<dl	9	<dl		<dl	<dl	<dl	4	<dl	
252	5/6/2009	7	M	3	216	107	<dl	<dl	<dl	<dl	<dl		<dl	<dl	trace	trace	<dl	
151	5/6/2009	8	F	9	na	na	<dl	<dl	<dl	<dl	<dl		<dl	<dl	1.6	37	<dl	
141	5/6/2009	8	F	19	232	149	<dl	<dl	<dl	<dl	<dl	<dl	<dl	<dl	<dl	6.5	<dl	<dl
217	5/6/2009	8	F	2	186	76	<dl	<dl					<dl	<dl				
215	5/6/2009	8	F	9	225	na	<dl	<dl					<dl	<dl				
219	5/7/2009	9	F	14	255	203	<dl	<dl	<dl	5	<dl		<dl	<dl	4.6	36	2.2	
254	5/7/2009	9	M	>17	266	247		<dl	<dl	9	<dl			<dl	2.4	trace	<dl	
125	5/7/2009	11	F	11	257	194		<dl	<dl	32	<dl	<dl		<dl	1.4	15	<dl	<dl
256	5/7/2009	11	M	2	210	106		<dl	<dl					<dl	trace			
198	5/7/2009	11	M	13	252	226		<dl	<dl		<dl			<dl	<dl		<dl	

### Brevetoxin extraction and analysis

Urine samples were centrifuged at 13000× *g* for 10 min at 25°C, supernatant removed, and 0.45 µm filtered prior to analysis. Fish viscera, dolphin feces and gastric samples were homogenized and extracted in acetone (3 volumes) four times, filtered via a 1 or 0.2 µm Pall syringe filter, dried under nitrogen gas, resuspended in 80% aqueous methanol (30 mL), twice solvent partitioned with hexane (30 mL), and the methanolic layer collected, dried under nitrogen gas and resuspended in 100% methanol (0.5 or 5 mL, depending on the weight of the original sample). Brevetoxins were extracted from blood cards using a phosphate buffered saline (94%)/methanol (6%) solvent (0.8 mL), followed by protein precipitation in acetonitrile (2.4 mL) [47]. Briefly, samples were centrifuged at 4000× *g* for 15 min at 4°C and the supernatant was collected. Plankton filter samples were collected by filtering ∼250 mL of seawater through Whatman GF/F filters and storing frozen at −20°C until analysis. Filters were twice extracted overnight with 2.5 mL methanol (100%) and vortexing with five short pulses [48] prior to centrifugation for 30 sec at 12000× *g* through NanoSep MF (0.2 µm) spin columns. All extracts were stored at −20°C until analysis.

The brevetoxin ELISA determined the presence of brevetoxin and brevetoxin-like compounds based on cross-reactivity with an antibody in a 96-well direct ELISA format [47]. In order to eliminate matrix effects, minimum assay dilutions for each sample type were: blood (1∶10), serum (1∶100), gastric fluid (1∶50), feces (1∶50), and fish viscera (1∶50). Sample calibration was performed using a brevetoxin-3 standard curve and non-linear regression analysis. The limits of detection (LOD) were: blood (0.2 ng/mL), serum (9 ng/mL), gastric fluid (2.7 ng/mL), feces (0.8 ng/g), and fish viscera (between 0.8 and 4.1 ng/g).

The brevetoxin radioimmunoassay (RIA) determined the presence of brevetoxin and brevetoxin-like compounds based on a sheep antiserum prepared against a brevetoxin-2 conjugate [49], [50], [51]. The RIA measured the competition between radiolabeled brevetoxin-3 (^3^H-brevetoxin-3) and unknown samples for the anti-brevetoxin-2 antiserum. Antibodies were filtered onto 25 mm glass fiber filters and the radioactivity of each filter was measured to determine the amount of brevetoxin-3 equivalents in each sample. The LODs were: blood (2.3 ng/mL), gastric fluid (between 0.5 and 3.5 ng/mL), feces (between 0.5 and 3.5 ng/g), and fish viscera (between 0.7 and 3.5 ng/g).

The brevetoxin receptor binding assay (RBA) measured competition between radiolabeled brevetoxin-3 (^3^H-brevetoxin-3) and unknown samples for the voltage-gated sodium channel in a rat brain crude membrane preparation, the pharmacological target of brevetoxins, to determine the total brevetoxin-3 equivalent activity of the sample. Details of this assay are described by Van Dolah *et al.* [52]. The LOD for feces samples in this assay was ∼100 ng brevetoxin-3-equiv./g.

The brevetoxin LC-MS/MS method measures the unambiguous structures of brevetoxin based on size, column retention and fragmentation patterns. Brevetoxin liquid chromatography (LC) separations were performed on a Luna C8(2) 150×2 mm column using an Agilent Technologies Model 1100 LC system. Mobile phase consisted of water (A) and acetonitrile (B), with 0.1% acetic acid additive. LC gradient: 2 min 35% B, linear gradient to 80% at 30 min, 95% B at 35 min, held at 95% B for 7 min, returned to 35% B at 43 min, and held for 7 min before the next injection. The mobile phase flow rate was 0.2 mL/min. The elutant from LC was analyzed by an Applied Biosystems/MDS Sciex 4000 QTRAP hybrid triple quadrupole/linear ion trap mass spectrometer equipped with a Turbo V™ source (Applied Biosystems, Foster City, CA, USA). The detection of brevetoxin congeners and metabolites by mass spectrometry was achieved by multiple reaction monitoring (MRM) and selected ion monitoring (SIM) [53], [54]. The following brevetoxin congeners and their derivatives were analyzed by comparison to commercial and/or in house derivatized standards: brevetoxin-1, -2, -3, -7, -9, oxidized brevetoxin-2, open A-ring brevetoxin-2, -3, -7, oxidized brevetoxin-2, cysteine-brevetoxin-A(B) and its sulfoxide, open-A ring cysteine-brevetoxin-B, and glutathione-brevetoxin-A(B). S/N ratio was about 26 for brevetoxin-3 standard at 1 ng/mL, 8 for brevetoxin-7 at 5 ng/mL, 54 for open-A ring brevetoxin-3 at 5 ng/mL, 76 for cysteine-brevetoxin-B at 10 ng/mL, and 17 for cysteine-brevetoxin-B sulfoxide at 10 ng/mL. The LODs for brevetoxin in seawater were 0.01 µg/L and 1 ng/mL urine.

### Domoic acid extraction and analysis

Dolphin urine and serum samples were centrifuged at 12000× *g* through a 0.22 µm filter column prior to analysis. Blood card samples (100 µL whole blood) were extracted for DA using a water (60%)/methanol (40%) solution (2.0 ml) according to the methods of Maucher and Ramsdell [55]. Briefly, samples were sonicated then extracted for 12 h at 4°C. Extracts were dried under nitrogen gas and resuspended in 10 mM PBS/0.05 Tween (100 uL). Gastric and feces samples were diluted 1∶4 with aqueous methanol (50%) prior to centrifugation for 10 min at 3000× *g* followed by filtration through a Nanosep (0.45 µm) syringe filter. Fish viscera were dissected from whole fish, homogenized and diluted 1∶4 with aqueous methanol (50%) prior to centrifugation for 10 min at 3000× *g* followed by filtration through a glass fiber filter (1 µm) followed by a Nanosep (0.45 µm) syringe filter. Fish were not allowed to thaw before the whole viscera was removed, in order to avoid leakage of DA from the digestive tract into the adjacent tissues [56]. Plankton filter samples were processed by filtering ∼250 mL of surface seawater through Watman GF/F filters and storing frozen at −20°C until extraction. Filters were extracted with 5 mL aqueous methanol (10%) prior to centrifugation for 1 min at 6000× *g* followed by filtration through a 0.22 µm syringe filter. All extracts were stored at −20°C until analysis.

A direct competitive DA enzyme-linked immunosorbent assay (ELISA) from Biosense Laboratories (Bergen, Norway) was used to screen the dolphin serum, urine, and blood card extracts [55]. This assay measures DA in a sample through its competition with DA coated onto microplate wells for anti-DA antibodies in solution. Blood card extracts were diluted 1∶10, serum samples were diluted 1∶100, and urine diluted 1∶200 prior to analysis. The limit of detection (LOD) of this assay was 0.12 ng/mL blood, 1.2 ng/mL serum, and 1.0 ng/mL urine.

Selected samples were analyzed for the presence of DA using tandem mass spectrometry coupled with liquid chromatographic separation (LC-MS/MS) [57]. This method utilized reverse phase chromatography, using an Agilent 1100 HPLC coupled to an Applied Biosystems/MDS Sciex API-4000 triple quadrupole mass spectrometer equipped with a Turbo V™ source (Applied Biosystems, Foster City, CA, USA). Chromatographic separation was performed on a Luna C18(2), 5 µm, 150×2 mm column (Phenomenex, Torrance, CA, USA). Mobile phase consisted of water and acetonitrile (ACN) in a binary system, with 0.1% formic acid as an additive. The elution gradient was: 3 min of 5% ACN, with a linear gradient to 40% ACN at 16 min, 95% ACN at 18 min, held for 5 min, then returned to initial conditions at 24 min and held for 5 min before the next injection. To reduce mass spectrometer contamination, a diverter valve was used to switch the LC eluant to the waste container except for the 6 minute window of LC eluant bracketing the retention time for DA that was sent to the MS. Retention time of DA in samples was determined based on the retention time observed with a certified reference standard (NRC Canada, Halifax, Canada). The detection of domoic acid by MS was achieved by multiple reaction monitoring (MRM) method with Turbo ion spray interface in positive ion mode. Four MRM transitions from protonated domoic acid were monitored: *m/z* 312→266, *m/z* 312→248, *m/z* 312→193, and *m/z* 312→161. The limits of quantification (LOQ) for this method were ∼0.5 ng/mL urine, 2 ng/mL gastric fluid, 0.01 µg/L seawater, and 2 ng/g dolphin feces or fish viscera, with a signal to noise ratio above ten.

### Statistical Analysis

Statistical analysis was conducted to examine the relationship between toxin (brevetoxin and DA) concentration and 16 hematologic and biochemical parameters. Hematology and blood chemistry data were categorized into 3 panels of interest: Red blood cell (RBC) indices, white blood cell (WBC) differential, and liver and/or kidney associated enzymes. A generalized linear model (GLM) was applied to examine the effect of toxin concentration for each of the three panels. Each panel was first analyzed using a multivariate GLM, and when the multivariate F-statistic (Wilks lambda) was significant (p<0.05), univariate GLMs (F-test) were conducted independently for each health parameter in the panel. Age was included as a covariate for the WBC differential and liver/kidney enzymes; sex was included as a covariate for RBC indices. The inclusion of covariates was based on previous examinations of factors influencing hematology and blood chemistry parameters [45]. P-plots were examined to assess normality of residuals, and variables were log-transformed when necessary (specifically for neutrophil, monocyte, lymphocyte and eosinophil counts) to meet model assumptions. Toxin concentrations from both urine and feces were included but in separate analyses.

## Results

**Figure 2:**
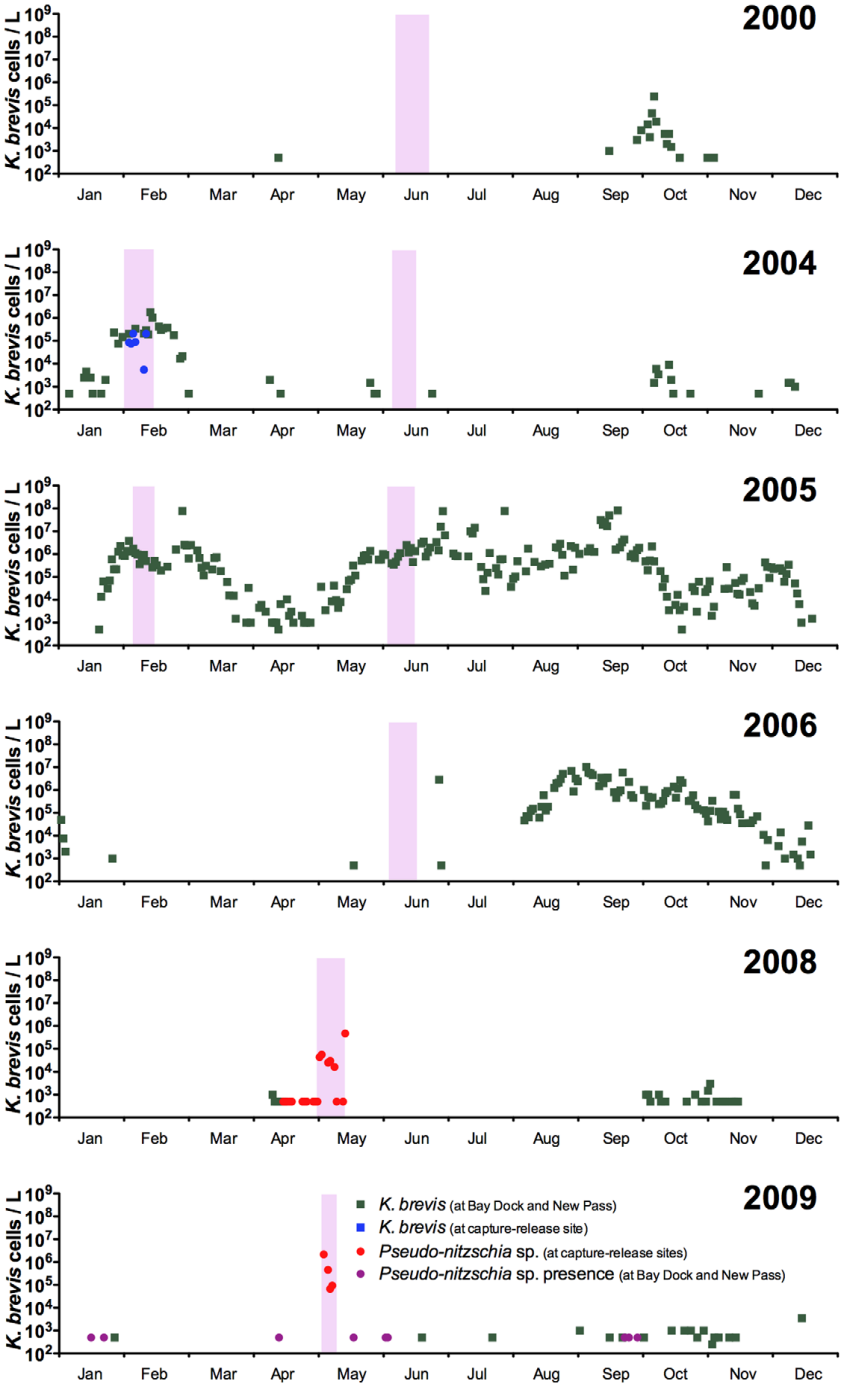
Representative *K. brevis* and *Pseudo-nitzschia* sp. cell count data for 2000 to 2009 in Sarasota Bay, Florida USA. Shaded regions represent the times during which dolphin health assessments occurred and samples were obtained for analyses.

In the Sarasota Bay region between 2000 and 2009, *K. brevis* cell densities varied between below the detection limit (1000 cells/L) to a maximum of 8×10^7^ cells/L (Figure 2). In each of these years, with the exception of 2008 and 2009, *K. brevis* cell densities exceeded 10^5^ cells/L. On three occasions, dolphin health assessments corresponded to periods in the bay when *K. brevis* cell counts were greater than 10^5^ cells/L (Feb 2004, Feb 2005, June 2005), whereas the remaining four health assessments occurred during periods when *K. brevis* was below the detection limit (June 2000, June 2004, June 2006, May 2008, May 2009). Unfortunately, *K. brevis* cell counts were not obtained at each set site during the health assessments. In May 2008 and 2009, seawater samples were analyzed for brevetoxin but all samples were below the limit of detection (<dl) of ∼0.01 µg/L (data not shown).

**Figure 3:**
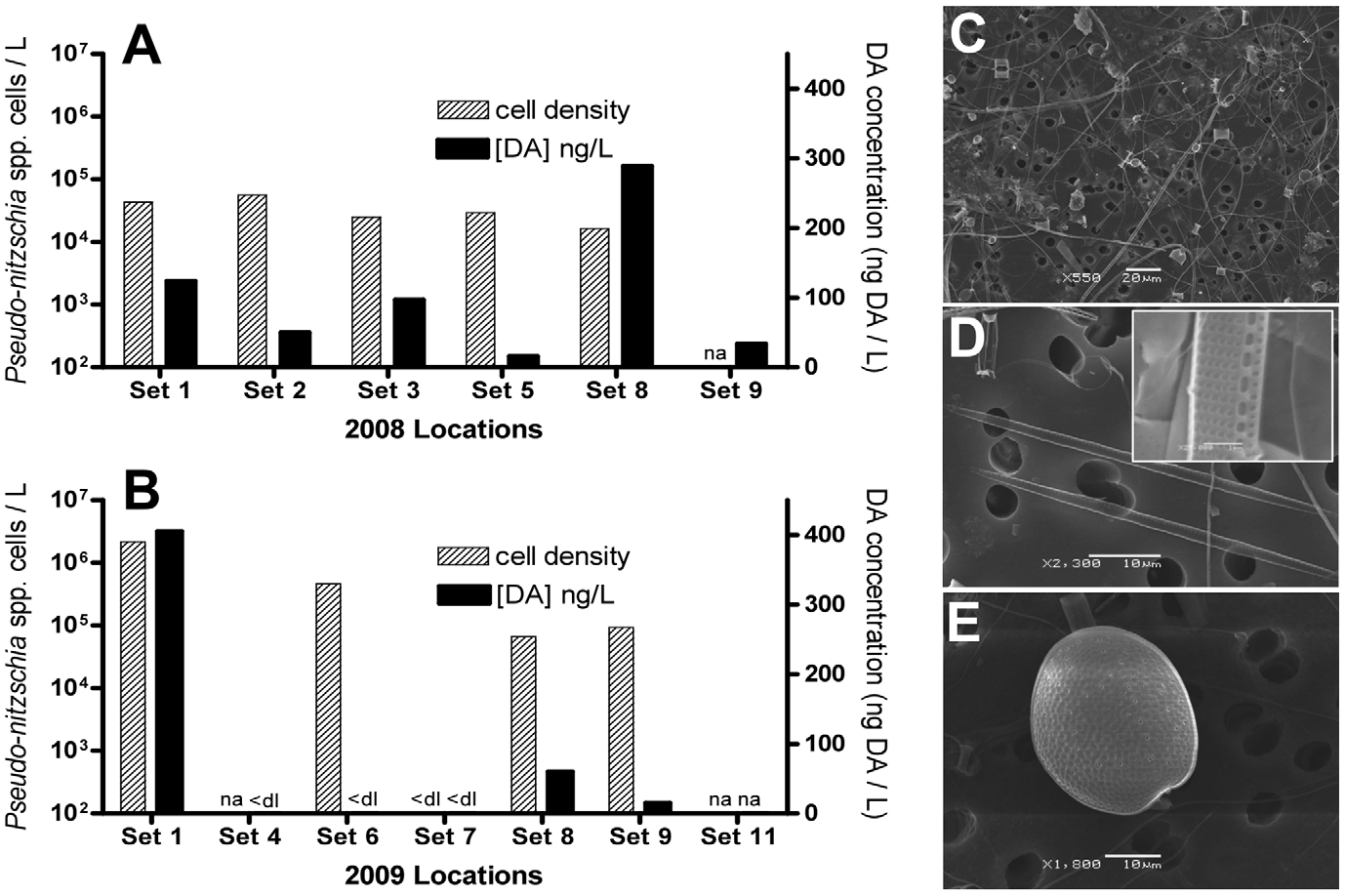
*Pseudo-nitzschia* spp. and domoic acid in Sarasota Bay, Florida, USA. Cell densities of *Pseudo-nitzschia* spp. and domoic acid water concentrations for various (A) 2008 and (B) 2009 set site locations. Scanning electron micrographs of three samples illustrating the presence of potential HAB species: C) A mixed assemblage of phytoplankton cells including *Chatocerous* spp. and *Pseudo-nitzschia* spp. from 2008 set site 8, D) *P. pseudodelicatissima* cells from 2008 sets sites 2, 5, and 8, and E) *Prorocentrum compressum* from 2008 set site 2. Note: Presence of *P. pseudodelicatissima* was also confirmed in 2008 set sites 8 and 2. ‘na’ represents ‘not available’ and ‘<dl’ represents ‘below detection limit’.

Prior to 2008, there were essentially no data available in the Sarasota Bay area on diatom presence, specifically *Pseudo-nitzschia* spp. However, in mid-April just prior to the 2008 dolphin health assessment, a *Pseudo-nitzschia* bloom occurred in the southern region of Sarasota Bay (Bay Dock, New Pass, Longboat Key, and Lido Key) (Figure 2) close to Mote Marine Laboratory with at least one sample containing over 3×10^6^ cells/L (Valeriy Palubok, pers. comm.). Not constrained to just the southern region of the bay, water samples from each set site all contained *Pseudo-nitzschia* densities in excess of 10^4^ cells/L (Figure 3A). These same water samples were used to positively identify the *Pseudo-nitzschia* cells as *P. pseudodelicatissima* by SEM (Figures 3C and 3D). Water samples containing *P. pseudodelicatissima* were confirmed in set sites 2, 5, and 8. Other harmful algal bloom species identified were *Chaetoceros* spp. (Figure 3C) and *Prorocentrum compressum* (Figure 3E). All seawater samples (n = 6) collected from each set site (Figure 1A) during the May 2008 health assessment were confirmed positive for DA with a mean particulate concentration of 101.7±40.9 ng/L (mean ± SE; range: 16–289 ng/L) (Figure 3A). These corresponded to cellular DA quotas of 5.15±3.17 pg/cell (mean ± SE; range: 0.54–17.57 pg/cell). Samples from set site 8, located at the mouth of Palma Sola Bay just north of Sarasota Bay, had the highest DA particulate and cell quota concentrations. Samples from set site 9 were compromised during the cell count procedure and not included.

Throughout much of 2009, low to moderate densities of *Pseudo-nitzschia* cells were observed at New Pass and Bay Dock but not quantified (Figure 2). During the May 2009 health assessment, *Pseudo-nitzschia* cells were observed at set sites 1, 6, 8, and 9 ranging in densities between 6.6×10^4^ to 2.2×10^6^ cells/L with set sites 1, 6, and 8 in the northern region of the bay and set site 9 in the southern end (Figure 3B). Mean particulate domoic acid concentrations were 80±65.7 ng/L (mean ± SE; range: <dl-405 ng/L) with three out of the six sites testing positive for DA (set sites 1, 8, and 9) (Figure 3B). Samples from set sites 1 and 8 were taken three days apart in Palma Sola Bay (northern region of the greater Sarasota Bay; see Figure 1B), whereas set site sample 9 was from the southern region of Sarasota Bay; suggesting patchy domoic acid distributions. These toxin concentrations result in DA cellular quotas of 0.42±0.23 pg/cell (mean ± range). At set sites 6 and 8, cells densities of *Pseudo-nitzschia* were in excess of 6.6×10^4^ cell/L but DA concentrations were <LOD.

Viscera from seven different fish species (striped mullet, pigfish, pinfish, striped mojarra, scaled sardines, mangrove snapper, sheepshead) collected in the Sarasota region in 2008 and/or 2009 were analyzed for both brevetoxin and DA concentrations (Table 2). Based on mean weight and calculated or measured standard length, all fish were determined to be adults (www.fishbase.org). In 2008, with the exception of the striped mojarra that were collected by the Chicago Zoological Society, all other fish were collected at set site 1 (Figure 1B). Mean brevetoxin concentrations for these species (striped mullet, pigfish, pinfish, striped mojarra, scaled sardines) ranged between 22 and 46 ng/g viscera and mean DA concentrations ranged between 39 and 440 ng/g viscera. Fish specimens that were of sufficient size (i.e., striped mullet, pinfish, striped mojarra; n = 15 total) for analysis of both toxins from the same homogenized tissues all tested positive for both brevetoxin (8–60 µg/g) and DA (9–171 µg/g). In 2009, striped mullet, pinfish, mangrove snapper, and sheepshead were caught over a two-day period in Sarasota Bay. Brevetoxin concentrations ranged between 3 and >20.3 ng/g viscera and DA concentrations ranged between <dl and 150.8 ng/g viscera. Although all individual fish tested positive for brevetoxin by ELISA and/or LC-MS/MS, only the striped mullet concurrently tested positive for DA as well.

**Table 2:** Concentrations of brevetoxin and domoic acid found in viscera of various fish species in Sarasota Bay, Florida, USA during the May 2008 and May 2009 dolphin health assessments.

Capture Date	Common Name	Genus species	Total No. Fish	Total Fish Weight (g)	Visceral Weight (g)	Brevetoxin (ng/g viscera)	Domoic acid (ng/g viscera)
5/1/2008	Striped mullet	*Mugil cephalus*	5	386±49	72±15	22±4 (n = 5*)	90±29 (n = 5*)
5/1/2008	Pinfish	*Lagodon rhomboides*	5	37±4	4.9±0.5	24±5 (n = 5*)	39±16 (n = 5*)
5/1/2008	Pigfish	*Orthopristis chrysoptera*	7	4.3±0.2	0.28±0.03	43±20 (n = 2)	76±21 (n = 5)
5/7/2008	Striped mojarra	*Eugerres plumieri*	5	151±42	18±4	45±6 (n = 5*)	65±15 (n = 5*)
5/1/2008	Scaled sardines	*Harengula jaguana*	10	14±0.7	3.5±0.6	46±4 (n = 5)	440±92 (n = 5)
5/5/2009	Striped mullet	*Mugil cephalus*	3	619±65.4	72.7±5.0	all >20.3 (n = 3*)	54±4 (n = 3*)
5/5/2009	Pinfish	*Lagodon rhomboides*	3	86±1.9	9.3±1.2	all >7 (n = 3*)	<dl (n = 3*)
5/7/2009	Mangrove snapper	*Lutjanus griseus*	2	147±5.5	7±0.2	3±1 (n = 2*)	<dl (n = 2*)
5/5/2009	Sheepshead	*Archosargus probatocephalus*	1	562	45.8	10 (n = 1*)	<dl (n = 1*)

Note: Data shown are mean ± SE (or ± range where n = 2). An asterisk (*) represents the same fish specimens used for both toxin analyses.

**Figure 4:**
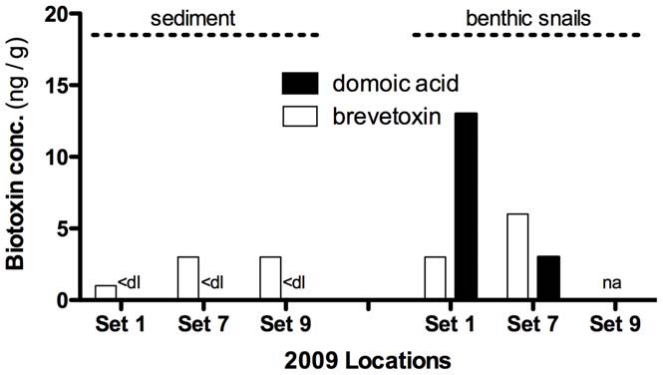
Domoic acid and brevetoxin in Sarasota Bay, FL sediment and benthic snails. Various samples for biotoxin analysis were collected in May 2009 that correspond to dolphin sampling set sites (“Sets”). Note: ‘na’ represents ‘not available’ and ‘<dl’ represents ‘below detection limit’.

During the 2009 health assessment event, benthic samples were taken for biotoxin analysis. All sediment samples (n = 3 total) collected from set sites 1, 7, and 9 were negative for DA, but positive for brevetoxin (by ELISA) ranging in concentration from 1–3 ng/g (Figure 4). At two sites, brevetoxin was identified at low concentrations in benthic snails (3–6 ng/g whole tissue) (ELISA with LC-MS/MS confirmation) with the concurrent presence of domoic acid (range: 3–13 ng/g tissue).

**Figure 5:**
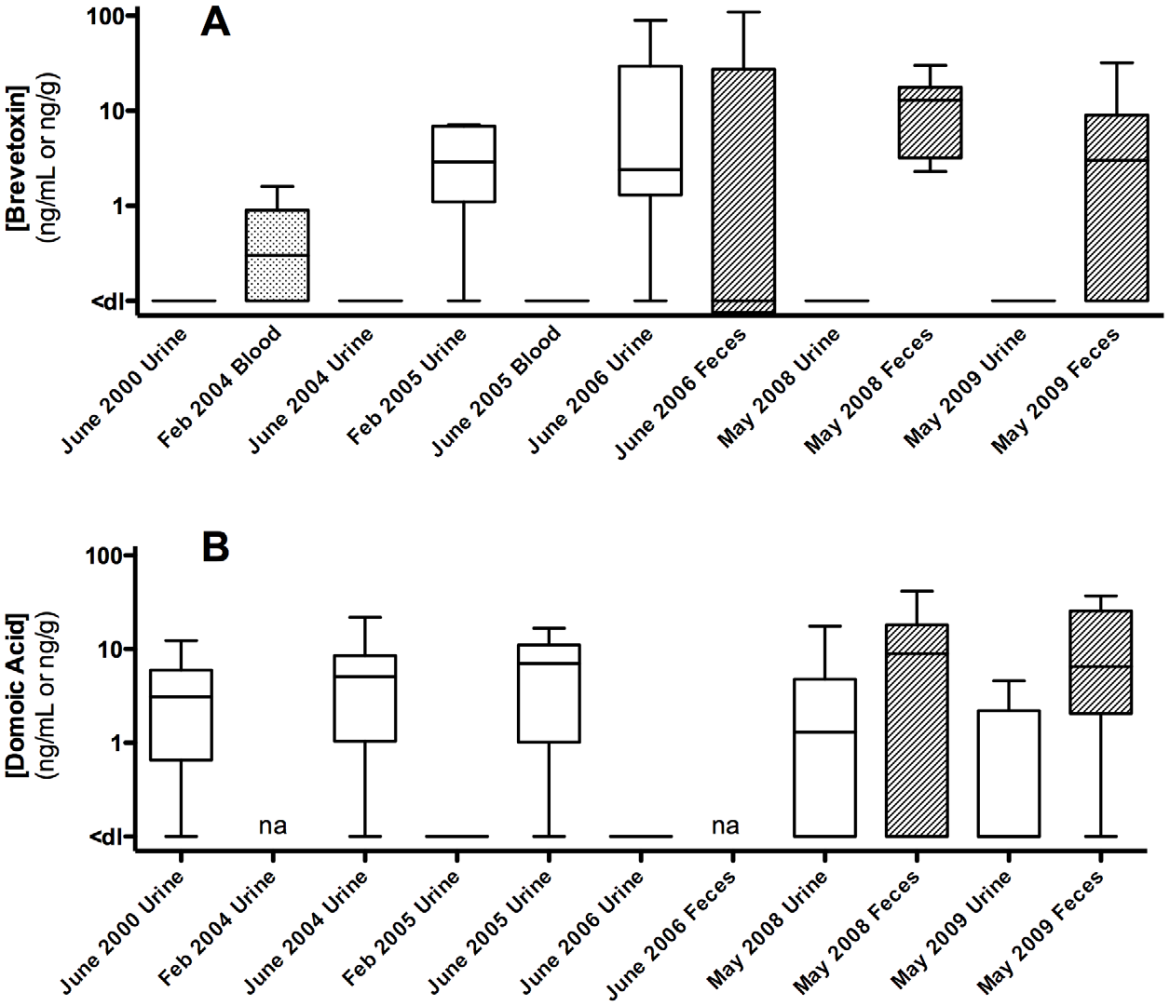
Biotoxin concentrations in urine, blood, and/or feces of Sarasota Bay, Florida dolphins between 2000–2009. (A) Brevetoxin was analyzed by RIA, ELISA, RBA, and/or LC/MS, and (B) domoic acid was analyzed by ELISA and/or LC/MS. Brevetoxin values are reported in ng PbTx-3 equiv./g or/mL. Data are median, quartile, and minimum/maximum values indicated by the midline, box, and whisker lines; respectively. Raw data are available in Table S1 and Table S2. Note: ‘na’ denotes samples not available.

During the May 2008 health assessment, several dolphin samples (blood, serum, urine, feces, gastric fluid) from 13 animals were obtained for biotoxin analysis. All feces (100%; 6/6) and most gastric fluid (92%; 11/12) samples tested positive for brevetoxin (2.3–30 ng/g or ng/mL) and a majority of urine (58%; 7/12) and feces (67%; 4/6) samples tested positive for DA (up to 17.6 ng/mL and 41.5 ng/g; respectively) (Table 1, Figure 5, and Table S1). Feces samples contained the highest concentrations of both biotoxins (brevetoxin: 2.3–30 ng/g; DA: <dl-41.5 ng/g) relative to other samples. Only one gastric fluid sample tested positive for DA (8%; 1/12) and all blood and serum samples were below the detection limit (<dl) for both toxins. With the exception of FB175 where only blood and serum samples were available, 7 of the remaining 12 dolphins (58%) concurrently contained both brevetoxin and DA in at least one sample type (urine, feces, and/or gastric fluid).

During the May 2009 health assessment, several dolphin tissue/fluid samples (blood, serum, urine, feces, gastric fluid, milk) from 14 animals were obtained for biotoxin analysis. Many feces (67%; 6/9) samples tested positive for brevetoxin (<dl-32 ng/g) and a majority of urine (67%; 8/12) and feces (100%; 9/9) samples tested positive for DA (up to 4.6 ng/mL and 36 ng/g; respectively) (Table 1, Figure 5, and Table S1). Only one gastric fluid sample tested positive for DA (9%; 1/11) and all blood, serum and milk samples were below the detection limit (<dl) for both toxins. Six out of the 14 dolphins (43%) concurrently contained both brevetoxin and DA in at least one sample type (most often feces samples).

Archived samples from dolphin health assessments in the Sarasota Bay area dating back to June 2000 (6 additional health assessments) were obtained and analyzed for both brevetoxin and DA (Figure 5, Table S1, and Table S2). All blood and serum samples were negative for brevetoxin and DA except for several brevetoxin-positive blood samples from February 2004. Nine out of 17 blood samples from February 2004 were positive for brevetoxin (53%; up to 1.3 ng/mL). Brevetoxin was detected in urine of animals sampled in February 2004 (1/1), February 2005 (4/5) and June 2006 (12/13) at concentrations up to 0.53, 7.1, 89.7 ng/mL; respectively, but was not detected in urine from animals in June 2000 (0/1) and June 2004 (0/9). Similar to May 2008, feces samples from June 2006 contained some of the highest brevetoxin concentrations observed (up to 101 ng brevetoxin-3 equiv./g). DA was detected in many of the urine samples collected in June 2000 (76%; 13/17), June 2004 (75%; 9/12), and June 2005 (83%; 5/6) up to concentrations of 12.3, 21.8, 16.7 ng/mL; respectively, but was not detected in urine samples from February 2005 (0/4) and June 2006 (0/15). Out of the 118 animals tested for brevetoxin over the ten-year period (including individual animals that were repeat samplings in different years), 43 were positive for brevetoxin (36%) whereas 44 out of 83 (53%) animals tested for DA were positive.

**Figure 6:**
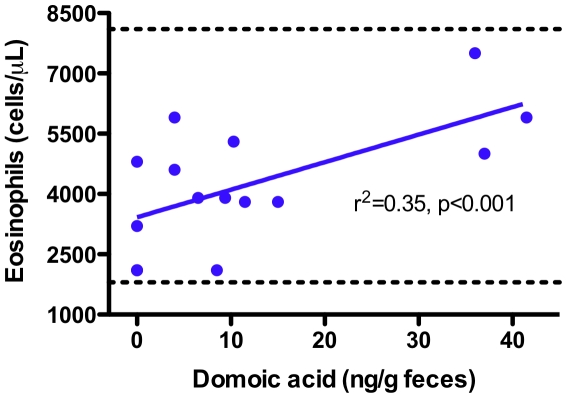
Domoic acid concentration in feces versus eosinophil count. Dashed green lines represent published reference thresholds [45]. Regression analysis indicated a statistically significant increase in eosinophil count (p<0.001), although none of the measured values were outside of established reference thresholds.

Three animals in particular were exposed to brevetoxin on two separate occasions (FB118, FB133 and FB188) while two individuals were exposed to DA on two separate occasions (FB11 and FB92) with a third animal exposed to DA three times (FB155) (Table S2). Of the eight health assessments analyzed within this study, five of these health assessments had several animals that tested positive for brevetoxin and five of these health assessments had several animals that tested positive for DA (Figure 5, Table S1, and Table S2). Over the course of this study, 13 animals (all from 2008 and 2009) were exposed to both brevetoxin and DA concurrently. Multivariate GLMs for both urine and fecal DA concentration (Table 3) showed a significant effect (p<0.05) on WBC differential and specifically on eosinophil count (urine p = 0.031, fecal p<0.001) (Figure 6). In addition, the effect of DA concentration in feces was significant for total WBC (p = 0.001). The effect of DA concentration in urine was significant for two RBC indices: red blood cell count (p = 0.002) and mean corpuscular volume (p<0.001). However, DA concentration in feces did not have a significant effect on RBC indices (p = 0.838). Brevetoxin concentration did not have a significant effect on any of the health panel indices.

**Table 3:** Relationships between toxin concentrations and various health parameters examined by p-values generated from generalized linear model (GLM).

Health Parameters	Domoic Acid		Brevetoxin	
	Urine	Fecal	Urine	Fecal
**RBC Indices; Covariate: Sex**	**0.008**	**0.838**	**0.114**	**0.229**
Red blood cells (RBCs)	0.002	na	na	na
Hematocrit (Hct)	0.245	na	na	na
Hemoglobin (Hgb)	0.355	na	na	na
Mean corpuscular volume (MCV)	<0.001	na	na	na
**WBC Differential; Covariate: Age**	**<0.001**	**0.011**	**0.906**	**0.773**
Total white blood cells (WBCs)	0.113	0.001	na	na
Neutrophils	0.768	0.386	na	na
Lymphocytes	0.612	0.127	na	na
Monocytes	0.651	0.542	na	na
Eosinophils	0.031	<0.001	na	na
**Liver & Kidney Enzymes; Covariate: Age**	**0.733**	**0.191**	**0.055**	**0.773**
Blood urea nitrogen (BUN)	na	na	na	na
Creatinine	na	na	na	na
Alanine aminotransferase (ALT)	na	na	na	na
Aspartate aminotransferase (AST)	na	na	na	na
Gamma-glutamyl transferase (GGT)	na	na	na	na
Lactate dehydrogenase (LDH)	na	na	na	na
Creatine kinase (CK)	na	na	na	na

Note: Bolded data indicate results from multivariate F-test (Wilks lambda). When multivariate p-value was less than 0.05, univariate F-tests were conducted for each dependent (health) variable. ‘na’ denotes not available.

## Discussion

In this study we documented the repeated exposure of bottlenose dolphins in southwest Florida to two marine algal toxins, brevetoxin and DA, over a ten year period (2000–2009) and measured toxin concentrations in whole water samples as well as in benthic samples and potential prey fish species that likely serve as important vectors for toxin trophic transfer in this region. We are the first to positively identify a known DA-producing species of *Pseudo-nitzschia* (*P. pseudodelicatissima*) in Sarasota Bay, FL, and to our knowledge, this is the first published report to describe concurrent exposure of marine mammals to multiple algal toxins in this region.

### *Karenia brevis* blooms and brevetoxin

*K. brevis* blooms are a nearly annual event in the southwestern region of Florida. Since *K. brevis* blooms are often patchy in their spatial distribution, cell count data from two frequently sampled sites (New Pass and Bay Dock) located at the southern end of Sarasota Bay were used as a proxy for bloom activity within the area. Although these data correlate with the cell count data in the other parts of the bay, they cannot be used to accurately assess toxin exposure to the dolphins or their prey items. During the course of this study, every year except for 2008 and 2009 had *K. brevis* cell densities greater than 10^5^ cells/L. Most notable were 2005 and August through November 2006 when cell densities exceeded 10^5^ cells/L for months at a time. During this particular period there was an ongoing multi-species UME (dolphins, manatees and sea turtles) that began in September 2005 and continued to July 2006 (www.nmfs.noaa.gov/pr/health/mmume/). During the eight dolphin health assessments in southwestern Florida that were used in this study, three of them occurred when *K. brevis* cell densities were considered ‘bloom’ conditions (≥10^5^ cells/L), whereas during the remaining five assessments *K. brevis* cell densities were at levels below detection (<10^3^ cells/L).

The exposure of marine mammals from the Sarasota Bay region to brevetoxin is not unusual or unexpected considering the intense *K. brevis* blooms that have occurred in the area on a nearly annual basis. In total, over a third of the animals (43 out of 118; 36%) collected between 2000 and 2009 were brevetoxin-positive, at concentrations similar to those published by Fire *et al.* [37]. The occurrence of brevetoxin in dolphin samples often correlated directly with the presence (February 2004, February 2005) or absence (June 2000, June 2004) of *K. brevis*. However, there were three occasions where low to moderate levels of brevetoxin were observed in the animals (June 2006, May 2008, May 2009) even though there was an absence of *K. brevis*. In fact, for each of these occasions it had been over 4 months since blooms of *K. brevis* had been detected in the area. This was in contrast to a previous study of Sarasota dolphins that consistently found an absence of brevetoxin in live dolphins during non-bloom conditions [58]. Nonetheless, some of the highest brevetoxin concentrations observed in this study were from animal samples collected in June 2006 (urine and feces), and the highest proportion of brevetoxin-positive animals were from May 2008 (92% animals positive) followed by May 2009 (43%). However, it should be noted that more sample types were collected and tested during the May 2008 assessment relative to the other assessments. Nonetheless, there appeared to be additional factors contributing to brevetoxin trophic transfer and bottlenose dolphin exposure beyond the presence or absence of a concurrent *K. brevis* bloom. Although some dolphin populations are migratory in nature, the population within Sarasota Bay is quite endemic. The current year-round resident population of dolphins in Sarasota Bay spans five generations and includes two individuals initially identified in 1970–71 and still routinely observed in the Sarasota Bay region [59]. The distribution of resightings of Sarasota dolphins suggests long-term residency and a definitive localized home range to Sarasota Bay [60].

Although the ichthyotoxic effects of brevetoxin are still being described [13], [16], [61], [62], it has been shown that blooms of *K. brevis* reduce fish density and species richness in Sarasota Bay [40]. Finfish compose the majority of the diet of bottlenose dolphins from the eastern Gulf of Mexico [38], [39] and have been postulated to play a role in brevetoxin trophic transfer to these mammals. Undigested fish collected from the stomachs of dolphins that died during the 2004 Florida Panhandle UME supported the link between many species of planktivorous fish (presumably feeding on *K. brevis*) and dolphin mortalities [18]. A subsequent study in the same area has shown that over 40 species of live planktivorous, omnivorous, and piscivorous fish contained brevetoxin in their tissues; some for up to one year following the termination of a *K. brevis* bloom [16]. Adult striped mullet (benthic omnivores that can feed on decaying plant and algal material) [63] and scaled sardines (pelagic omnivores feeding at least partially on copepods) [64], [65] contained some of the highest brevetoxin values, particularly in the liver and gastrointestinal contents [16]. Similarly, viscera from many of the fish in Sarasota Bay that are known to be prey for resident dolphins [38] contained toxin concentrations ranging between 68 and 190 ng brevetoxin-3 equiv./g in the absence of *K. brevis* [17]. When *K. brevis* was present, brevetoxin concentrations were much higher (pinfish>pigfish>mullet), averaging between 81 and 1313 ng brevetoxin-3 equiv./g viscera. During controlled laboratory exposures of striped mullet to *K. brevis*, blood brevetoxin concentrations varied according to the *K. brevis*/brevetoxin exposure concentrations where maximal blood brevetoxin concentrations were ∼19 ng/mL at 8 to 12 hr post-exposure with a protracted retention time (T_1/2_) of over 11 days [66]. Studies subsequently performed with Atlantic menhaden (*Brevoortia tyrannus*) also demonstrated slow elimination rates with a half-life of 24 days [67]. Although in our study the sample size was relatively small (n = 1, 2 or 5 per fish species), these slow rates of elimination probably cannot account for the observation that brevetoxin was found in every fish species and each individual collected in May 2008 and May 2009, many months since the previous *K. brevis* bloom. As such, secondary routes of brevetoxin trophic transfer might have been involved in these protracted residency times of at least 5 months.

Omnivorous and piscivorous fish may also accumulate brevetoxins via indirect routes of trophic transfer [19]. As observed in St. Joseph Bay, Florida, non-planktivorous fish accumulate moderate to high concentrations of brevetoxin in their tissues, particularly the liver tissues of piscivorous fish such as red snapper (*Lutjanus campechanus*; up to 16483 ng brevetoxin-3 equiv./g) [16]. One trophic route was demonstrated in laboratory studies where pinfish and croakers (*M. undulates*) accumulated brevetoxin via feeding on contaminated shellfish [16]. Brevetoxin trophic transfer may also begin with herbivorous fish feeding on contaminated seagrass. In 2002, 34 manatees died due to brevetoxicosis during a UME in southwest Florida following consumption of brevetoxin-contaminated seagrass such as *Thalassia testudinum* [18]. Organisms feeding on (i.e., manatees, sea turtles) (D. Fauquier, pers. comm.) living within (i.e., macrobenthic epifauna) [68], or associated with (i.e., epiphytes, macroalgae) [69] seagrass may also be susceptible to brevetoxin exposure. In addition, a benthic brevetoxin sink [70] may play a role in toxin (re)cycling into benthic organisms or into the water column during resuspension events as moderate amounts of toxin can be found in coastal sediments at concentrations up to 9.7 ng brevetoxin/g dry sediment [71]. This finding is supported by the present study, which observed similar levels in sediment (1–3 ng/g) and benthic snails (3–6 ng/g) providing evidence that benthic brevetoxin is biologically available.

Based on the comparable concentrations of brevetoxin for each of the five fish species collected in May 2008 (22–46 ng brevetoxin equiv./g viscera), it appears that in the absence of any recent *K. brevis* bloom activity, these particular fish species pose an approximately equal degree of risk for bottlenose dolphins. This is unusual since the feeding behaviors of these fish species, all adults based on size, are quite distinct. Scaled sardines are the most likely to feed directly on *K. brevis*. Striped mullet feed on decaying plant detritus and sediment which may include epiphytic and benthic microalgae (such as dead or dying *K. brevis*), [63]. Pigfish, pinfish, and striped mojarra are more likely to be exposed to brevetoxin via indirect routes such as feeding on crustaceans, gastropods and/or polychaetes [64], [72]. Pinfish in particular are well known to associate with seagrass beds, and much of their diet can be composed of vegetation [73]. Interestingly, scaled sardines as well as other members of the clupeidae family (i.e., herrings, shads, sardines, anchovies, menhadens) were shown to be positively associated with *K. brevis* density while all other guilds significantly decreased during *K. brevis* bloom events in Sarasota Bay making clupeids potentially an even more vital dietary prey item for bottlenose dolphins as well as an effective vector for brevetoxin exposure [40]. The potential importance of striped mullet as a source for biotoxin trophic transfer the Sarasota Bay region was again identified in May 2009. Although all fish species tested were positive for brevetoxin (striped mullet, pinfish, mangrove snapper, sheepshead) (range: 3->20.3 ng/g), the striped mullet also concurrently contained DA (mean ± SE: 54±4 ng/g; n = 3). In the Florida Panhandle, scaled sardines accumulate very high concentrations of brevetoxin (>2400 ng/g liver and GI contents) [16] and are not as susceptible to the ichthyotoxic effects of *K. brevis*.

### *Pseudo-nitzschia* blooms and domoic acid

In mid-April 2008, a *Pseudo-nitzschia* bloom was observed in the southern region of Sarasota Bay with densities exceeding 1.5 million cells/L. *P. pseudodelicatissima* and DA were concurrently identified in samples collected from three set sites within Sarasota and Palma Sola Bays in early May 2008. Cell quotas for these samples (5.15 pg/cell) were similar to field estimates of *P. pseudodelicatissima* from Louisiana (4.82 pg/cell) [31] and *P. multiseries* from Texas (2.1 pg/cell) [29], but an order of magnitude lower than some *P. australis* cells from California (7–75 pg/cell) obtained during the 1998 sea lion UME [22]. In 2009, high densities of *Pseudo-nitzschia* spp. were observed in Palma Sola Bay (up to 2.2×10^6^ cells/L) with most samples in Sarasota Bay also containing significant densities of *Pseudo-nitzschia* (>10^4^ cells/L). However, DA concentrations in the surface water varied greatly (<dl to 405 ng/L) with low corresponding cell quotas (0.42 pg/cell). Although no ASP events have been documented in the Gulf of Mexico, clearly species of *Pseudo-nitzschia* have been established in the northern region for many years, and we have now demonstrated that toxigenic species of *Pseudo-nitzschia* are also present in southwestern Florida.

In light of the fact that prior to May 2008 there were no documented *Pseudo-nitzschia* blooms in southwestern Florida, this study has demonstrated that since at least June 2000, dolphins in this area have been exposed to DA. DA was detected in at least one animal during five out of the eight health assessments. Over our ten-year study period, more than half of the animals (44 out of 83; 53%) tested were positive for DA. Considering the intensity and frequency of *K. brevis* blooms, it seems unusual that there were a greater proportion of DA exposed animals than there were brevetoxin exposed animals (36%). The pharmacokinetic properties of DA in mammals (i.e., rats) suggest that relative to brevetoxin, DA is short-lived and quickly eliminated [74], [75]. For DA to be observed so often in southwestern Florida dolphins, DA-producing *Pseudo-nitzschia* spp. are likely present with greater frequency and abundance than previously believed or cryptic sources of DA (e.g., benthic) may re-emerge during non-bloom periods. In three animal samples collected in May 2009, milk from lactating females were all DA negative even in light of finding DA in corresponding feces of those same mothers. The absence of DA suggests that at that particular point in time, the potential for maternal transfer of DA was minimal.

The trophic transfer of DA has been well documented with growing evidence that both pelagic and benthic routes are important. In the Sarasota Bay region, all fish collected in 2008 (n = 5 individuals of 5 species) were positive for DA. In 2009, only the striped mullet were found to contain DA, whereas the other fish species (pinfish, mangrove snapper, sheepshead) were all negative. The benthic-feeding striped mullet (omnivore; *M. cephalus*) and scaled sardines (pelagic filter feeder; *Harengula jaguana*) contained the highest concentrations of DA, suggesting that toxin content via direct exposure was at least partially explained by the feeding behavior of these fish species. Although the DA concentrations observed in the scaled sardines were still 2 orders of magnitude less than what would be considered unfit for consumption by FDA guidelines (20 µg/g or 20 ppm) and 1–3 orders of magnitude less than levels observed in anchovies and sardines off the coast of California associated with UMEs (0.27–223 µg/g viscera or gut) [22], [23], it has been suggested that Gulf of Mexico clupeid species become even more important dolphin dietary items during and following *K. brevis* events [40]. This is postulated to occur as the abundance of the dolphin's normal primary prey items (i.e., pigfish, pinfish, mullet) become significantly reduced. As such, clupeids may play a significant role in the trophic transfer of both DA and brevetoxin to southwestern Florida dolphins.

Vertical fluxes of DA from blooms of DA-producing *Pseudo-nitzschia* blooms into sediments may in turn contaminate benthic organisms [70], [76] such as demersal fish [77], shellfish [27], and crustaceans [28]. Benthic recycling or reemergence of DA in the Sarasota Bay region may be a significant initiation point for new trophic transfer pathways exposing dolphins to this toxin. In this study, benthic snails collected from two sites contained 3–13 ng DA/g. On the west coast of the US, DA has been observed in other benthic organisms [77], [78] presumably following rapid downward transport of *Pseudo-nitzschia* cells and toxin from ongoing pelagic blooms [70] and provide new dynamic routes of exposure to marine mammals. Although sediments from Sarasota Bay were below the limit of detection, DA has been shown to adsorb to sediments. However, since the desorption rates of DA have been shown to be quite rapid (i.e., minutes), in turn maintaining equilibrium with the surrounding water [79], unless DA was continually supplied, DA's half-life in the sediment will be relatively short.

### Bottlenose dolphin health

Relative to brevetoxin concentrations reported for dolphins from UMEs occurring in other regions of Florida [18], the concentrations of brevetoxins observed in live Sarasota animals were generally 1–2 orders of magnitude less. No Sarasota Bay resident dolphins were known to have died as a result of exposure to brevetoxin in 2005, even in the midst of a UME that encompassed Sarasota Bay and surrounding waters. During these UMEs, dolphin stomach contents typically contained the highest concentrations of brevetoxin, ranging from <dl to >54000 ng/g (n = 53 animals) during the 2005/06 central west Florida UME (unpublished data) and 136 to 6176 ng/g (mean = 2126; n = 30 animals) during the 2004 Panhandle UME [18]. Similarly, marine mammals that have died during DA-related UMEs have had much higher concentrations of DA within their tissues relative to live Sarasota animals. In 1998, deceased California sea lions had in excess of 1 µg DA/mL feces (up to 182 µg/mL) and between 0.12 and 3.7 µg DA/mL urine; 6- to 40-fold higher than any concentration observed in this study. Nonetheless, the exposure of these animals to either DA or brevetoxin at low concentrations over long periods of time raises issues regarding potential chronic health effects.

In this study we examined the relationship between measured low-level biotoxin concentrations and panels of hematologic and biochemical parameters. No relationship was found between brevetoxin concentration and any of the health panels. However, a weak but statistically significant effect of DA concentration was determined for WBC differential; specifically there were increases in total WBC and eosinophil counts. The effect of DA on eosinophil counts was significant regardless of the matrix (urine or feces) from which DA was measured. Yet even the highest eosinophil count (7500 eosinophils/µL; DA concentration = 36.8 ng/g feces) was still lower than the established reference threshold (8100 eosinophils/µL) [45] suggesting that the low levels of DA are not perturbing hematological parameters outside of the “normal” biological range. Nonetheless, these findings were significant in that the results were consistent with prior studies in mammals. A relatively large percentage of Florida Panhandle dolphins (23%) that were exposed to DA (<dl - 201 ng/mL urine) had eosinophil counts that were well above the established reference threshold [46]. The study further found that the elevated eosinophils were associated with more sensitive indicators of functional immune status, such as decreased T-lymphocyte proliferation and increased neutrophil phagocytosis. Similarly, California sea lions with clinical symptoms of DA toxicity had elevated eosinophil counts [80], which is further supported by the observed immunomodulatory effects of DA on mammalian WBCs [81], [82]. As such, the results from this study support the hypothesis that DA caused an immunomodulatory response in these dolphins, although the implications for an individual survival outcome and/or a population level health effect require further elucidation.

Over the course of this study, 13 animals (14%) (all from 2008 and 2009) were concurrently exposed to both brevetoxin and DA. Although neither of the toxin concentrations approached concentrations observed in UME animals, the presence of more than one toxin in individual animals raises questions involving potential synergistic effects. Preliminary data investigating the effects of both DA and brevetoxin suggested that there are no synergistic effects of these toxins on mouse lethality following acute, single dose exposures (J. Naar, pers. comm.) or neuroblastoma cytotoxicity (Y. Bottein, pers. comm.). Nonetheless, stomach content, urine and/or blood samples collected from 9 stranded animals during the 2004 Panhandle UME all contained low concentrations of DA (<10 ng DA/g or mL) in addition to high concentrations of brevetoxin (M. Twiner, unpublished data. One possible scenario is that the presence of multiple toxins may first result in reduced health such as immunosuppression, which in turn makes these animals more sensitive to secondary stressors leading to death. In addition to well-described neurological toxicities of DA and brevetoxin, there is a growing amount of evidence that suggests that both DA [82], [83] and brevetoxin [84], [85] also have immunomodulatory effects that may aid in this sensitization. It has recently been shown UME bottlenose dolphins off the coast of Texas (USA) were concurrently exposed to both DA and okadaic acid [86] and that the endangered North Atlantic right whales (*Eubalaena glacialis*) are annually exposed to both DA and saxitoxin via consumption of contaminated copepods (*Calanus fimmarchicus*) [87] with postulated long-term effects on reproductive dysfunction.

It is generally acknowledged that there is an apparent increase in the incidence and intensity of HABs [10], [88] potentially influenced by natural and anthropogenic factors, including eutrophication [89] and global climate change [90]. If this apparent increase represents an actual increase in bloom events involving different HAB species producing unique toxic molecules, there will likely be a greater chance of concurrent exposure of aquatic organisms such as dolphins and humans to multiple HAB toxins. It is therefore imperative that future studies attempt to gain a better understanding of the potential effects of exposure to multiple toxins.

## Supporting Information

Table S1 **Concentration of brevetoxin (ng/mL or ng/g) in various animal samples.**
(PDF)

Table S2 **Concentration of domoic acid (ng/mL or ng/g) in various animal samples.**
(PDF)

## References

[1] Steidinger KA, Vargo GA, Tester PA, Tomas CR, Anderson DM, Cembella AD, Hallegraeff GM (1998). Bloom dynamics and physiology of *Gymnodinium breve* with emphasis on the Gulf of Mexico.. Physiological ecology of harmful algal blooms: NATO ASI series.

[2] Catterall WA, Risk M (1981). Toxin T4(6) from *Ptychodiscus brevis* (formerly *Gymnodinium breve*) enhances activation of voltage-sensitive sodium channels by veratridine.. Mol Pharmacol.

[3] Lin Y-Y, Risk M, Ray SM, Van Engen D, Clardy J (1981). Isolation and structure of brevetoxin B from the “red tide” dinoflagellate *Ptychodiscus brevis* (*Gymnodinium breve*).. J Am Chem Soc.

[4] Baden DG, Mende TJ, Poli MA, Block RE, Ragelis EP (1984). Toxins from Florida's red tide dinoflagellate *Ptychodiscus brevis*.. Seafood toxins: American Chemical Society.

[5] Poli MA, Mende TJ, Baden DG (1986). Brevetoxins, unique activators of voltage-sensitive sodium channels, bind to specific sites in rat brain synaptosomes.. Mol Pharmacol.

[6] Huang JM, Wu CH, Baden DG (1984). Depolarizing action of a red-tide dinoflagellate brevetoxin on axonal membranes.. J Pharmacol Exp Ther.

[7] Ramsdell JS, Botana LM (2008). The molecular and integrative basis to brevetoxin toxicity.. Seafood and Freshwater Toxins: Pharmacology, Physiology, and Detection.

[8] McFarren EF, Tanabe H, Silva FJ, Wilson WB, Campbell JE (1965). The occurrence of a ciguatera-like poison in oysters, clams, and *Gymnodinium breve* cultures.. Toxicon.

[9] Kirkpatrick B, Fleming LE, Squicciarini D, Backer LC, Clark R (2004). Literature review of Florida red tide: implications for human health effects.. Harmful Algae.

[10] Van Dolah FM (2000). Marine algal toxins: origins, health effects, and their increased occurrence.. Environmental Health Perspectives.

[11] Woodcock AH (1948). Note concerning human respiratory irritation associated with high concentrations of plankton and mass mortality of marine organisms.. J Mar Res.

[12] Asai JS, Krzanowski JJ, Lockey RF, Anderson WH, Martin DF (1984). Site of action of *Ptychodiscus brevis* toxin within parasympathetic axonal sodium channel h gate in airway smooth muscle.. J Allergy Clin Immunol.

[13] Steidinger K, Burklew MA, Ingle RM, Martin DF, Padilla GM (1972). The effects of *Gymnodinium breve* toxin on estuarine animals.. Marine Pharmacognosy.

[14] Bossart GD, Baden DG, Ewing RY, Roberts B, Wright SD (1998). Brevetoxicosis in manatees (*Trichechus manatus latirostris*) from the 1996 epizootic: gross, histologic and immunohistochemical features.. Toxicol Pathol.

[15] Mase B, Jones W, Ewing R, Bossart GD, Van Dolah FM Epizootic in bottlenose dolphins in the Florida panhandle: 1999–2000;.

[16] Naar JP, Flewelling LJ, Lenzi A, Abbott JP, Granholm A (2007). Brevetoxins, like ciguatoxins, are potent ichthyotoxic neurotoxins that accumulate in fish.. Toxicon.

[17] Fire SE, Flewelling LJ, Naar J, Twiner MJ, Henry MS (2008). Prevalence of brevetoxins in prey fish of bottlenose dolphins in Sarasota Bay, Florida.. Marine Ecology Progress Series.

[18] Flewelling LJ, Naar JP, Abbott JP, Baden DG, Barros NB (2005). Brevetoxicosis: red tides and marine mammal mortalities.. Nature.

[19] Landsberg JH, Flewelling LJ, Naar J (2009). *Karenia brevis* red tides, brevetoxins in the food web, and impacts on natural resources: Decadal advancements.. Harmful Algae.

[20] Wright JLC, Quilliam M (1989). The amnesic shellfish poisoning mystery.. Anal Chem.

[21] Work TM, Bar B, Beale AM, Fritz L, Quilliam M (1992). Epidemiology of domoic acid poisoning in brown pelican's (*Pelecanus occidentalis*) and Brandt's cormorants (*Phalacrocorax penicillatus*) in California.. J Zoo Wildl Med.

[22] Scholin CA, Gulland F, Doucette GJ, Benson S, Busman M (2000). Mortality of sea lions along the central California coast linked to a toxic diatom bloom.. Nature.

[23] Lefebvre KA, Powell CL, Busman M, Doucette GJ, Moeller PDR (1999). Detection of domoic acid in northern anchovies and california sea lions associated with an unusual mortality event.. Natural Toxins.

[24] Van Dolah FM, Reynolds JE, Perrin WF, Reeves RR, Montgomery S, Ragen TJ (2005). Effects of harmful algal blooms.. Marine Mammal Research: Conservation beyond crisis.

[25] Hampson D, Manalo J (1998). The activation of glutamate receptors by kainic acid and domoic acid.. Natural Toxins.

[26] Bargu S, Powell CL, Coale SL, Busman M, Doucette GJ (2002). Krill: a potential vector for domoic acid in marine food webs.. Mar Ecol Prog Ser.

[27] Blanco J, Acosta CP, Bermudez de la Puente M, Salgado C (2002). Depuration and anatomical distribution of the amnesic shellfish poisoning (ASP) toxin domoic acid in the king scallop *Pecten maximus*.. Aquatic Toxicol.

[28] Ferdin ME, Kvitek RG, Bretz CK, Powell CL, Doucette GJ (2002). *Emerita analoga* (Stimpson)–possible new indicator species for the phycotoxin domoic acid in California coastal waters.. Toxicon.

[29] Dickey RW, Fryxell GA, Granade HR, Roelke D (1992). Detection of the marine toxins okadaic acid and domoic acid in shellfish and phytoplankton in the Gulf of Mexico.. Toxicon.

[30] Fryxell GA, Reap ME, Valencic DL (1990). *Nitzschia pungens* Grunow f. *multiseries* Hasle: observations of a known neurotoxic diatom.. Nova Hedwigia (Beih).

[31] Parsons ML, Scholin CA, Miller PE, Doucette GJ, Powell CL (1999). *Pseudo-nitzschia* species (Bacillariophyceae) in Louisiana coastal water: Molecular probe field trials, genetic variability, and domoic acid analyses.. J Phycol.

[32] Pan Y, Parsons ML, Busman M, Moeller P, Dortch Q (2001). *Pseudo-nitzschia* sp. cf. *pseudodelicatissima*- a confirmed producer of domoic acid from the northern Gulf of Mexico.. Mar Ecol Prog Ser.

[33] Liefer JD, MacIntyre HL, Novoveská L, Smith WL, Dorsey CP (2009). Temporal and spatial variability in *Pseudo-nitzschia* spp. in Alabama coastal waters: A “hot spot” linked to submarine groundwater discharge?. Harmful Algae.

[34] Wells RS, Rhinehart HL, Hansen LJ, Sweeney JC, Townsend FI (2004). Bottlenose dolphins as marine ecosystem sentinels: Developing a health monitoring system.. EcoHealth.

[35] Schwacke LH, Voit EO, Hansen LJ, Wells RS, Mitchum GB (2002). Probabilistic risk assessment of reproductive effects of polychlorinated biphenyls on bottlenose dolphins (*Tursiops truncatus*) from the southeast United States coast.. Enviro Toxicol Chem.

[36] Wells RS, de Waal FBM, Tyack PL (2003). Dolphin social complexity: Lessons from long-term study and life history.. Animal Social Complexity: Intelligence, Culture, and Individualized Societies.

[37] Fire SE, Flewelling LJ, Wang Z, Naar J, Henry MS (2008). Florida red tide and brevetoxins: Association and exposure in live resident bottlenose dolphins (*Tursiops truncatus*) in the eastern Gulf of Mexico, U.S.A.. Marine Mammal Science.

[38] Barros NB, Wells RS (1998). Prey and feeding patterns of resident bottlenose dolphins (*Tursiops truncatus*) in Sarasota Bay, Florida.. Journal of Mammalogy.

[39] Barros NB, Odell DK, Leatherwood S, Reeves RR (1990). Food habits of bottlenose dolphins in the Southeastern United States.. The Bottlenose Dolphin.

[40] Gannon DP, Berens McCabe EJ, Camilleri SA, Gannon JG, Brueggen MK (2009). Effects of *Karenia brevis* harmful algal blooms on nearshore fish communities in southwest Florida.. Mar Ecol Prog Ser.

[41] Plakas SM, Wang Z, El Said KR, Jester ELE, Granade HR (2004). Brevetoxin metabolism and elimination in the Eastern oyster (*Crassostrea virginica*) after controlled exposures to *Karenia brevis*.. Toxicon.

[42] Utermöhl H (1958). Zur Vervolkommung der quantitativen phytoplankton.. Mitt Int Verein Limnol.

[43] Hasle GR, Sournia A (1978). The inverted microscope.. Phytoplankton Manual.

[44] Morton SL (1998). Morphology and toxicology of *Prorocentrum faustiae* sp. nov., a toxic species of non-planktonic dinoflagellates from Heron Island, Australia.. Botanica Marina.

[45] Schwacke LH, Hall AJ, Townsend FI, Wells RS, Hansen LJ (2009). Hematologic and serum biochemical reference intervals for free-ranging common bottlenose dolphins (*Tursiops truncatus*) and variation in the distributions of clinicopathologic values related to geographic sampling site.. Am J Vet Res.

[46] Schwacke LH, Twiner MJ, De Guise S, Balmer BC, Wells RS (2010). Eosinophilia and biotoxin exposure in bottlenose dolphins (*Tursiops truncatus*) from a coastal area impacted by repeated mortality events.. Enviro Res.

[47] Maucher JM, Briggs LM, Podmore C, Ramsdell JS (2007). Optimization of blood collection card method/enzyme-linked immunoassay for monitoring exposure of bottlenose dolphin to brevetoxin-producing red tides.. Environ Sci Technol.

[48] Roth PB, Twiner MJ, Wang Z, Bottein Dechraoui M-Y, Doucette GJ (2007). Brevetoxin (PbTx) dynamics following lysis of *Karenia brevis* by algicidal bacteria: Toxin size fractions and characterization of open A-ring PbTx hydrolytics.. Toxicon.

[49] Woofter RT, Bottein Dechraoui M-Y, Garthwaite I, Towers NR, Gordon CJ (2003). Measurement of brevetoxin levels by radioimmunoassay of blood collection cards after acute, long-term, and low-dose exposure in mice.. Environ Health Persp.

[50] Poli MA, Rein KS, Baden DG (1995). Radioimmunoassay for PbTx-2-type brevetoxins: epitope specificity of two anti-PbTx sera.. J AOAC Int.

[51] Poli MA, Musser S, Dickey RW, Eilers PP, Hall S (2000). Neurotoxic shellfish poisoning and brevetoxin metabolites: a case study from Florida.. Toxicon.

[52] Van Dolah FM, Finley EL, Haynes BL, Doucette GJ, Moeller PD (1994). Development of rapid and sensitive high throughput pharmacologic assays for marine phycotoxins.. Natural Toxins.

[53] Plakas SM, Jester ELE, El Said KR, Granade HR, Abraham A (2008). Monitoring of brevetoxins in the *Karenia brevis* bloom-exposed Eastern oyster (*Crassostrea virginica*).. Toxicon.

[54] Wang Z, Plakas SM, El Said KR, Jester ELE, Granade HR (2004). LC/MS analysis of brevetoxin metabolites in the Eastern oyster (*Crassostrea virginica*).. Toxicon.

[55] Maucher J, Ramsdell J (2005). Ultrasensitive detection of domoic acid in mouse blood by competitive ELISA using blood collection cards.. Toxicon.

[56] Lefebvre KA, Dovel SL, Silver MW (2001). Tissue distribution and neurotoxic effects of domoic acid in a prominent vector species, the northern anchovy *Engraulis mordax*.. Mar Biol.

[57] Wang Z, King KL, Ramsdell JS, Doucette GJ (2007). Determination of domoic acid in seawater and phytoplankton by liquid chromatography-tandem mass spectrometry.. Journal of Chromatography A.

[58] Fire SE, Fauquier D, Flewelling LJ, Henry MS, Naar J (2007). Brevetoxin exposure in bottlenose dolphins (*Tursiops turncatus)* associated with *Karenia brevis* blooms in Sarasota Bay, Florida.. Mar Biol.

[59] Wells RS, Tornero V, Borrell A, Aguilar A, Rowles TK (2005). Integrating life-history and reproductive success data to examine potential relationships with organochlorine compounds for bottlenose dolphin (*Tursiops truncatus*) in Sarasota Bay, FL.. Sci Total Environ.

[60] Wells RS, Scott MD, Hammond PS, Mizroch SA, Donovan GP (1990). Estimating bottlenose dolphin population parameters from individual identification and capture release techniques.. Individual Recognition of Cetaceans: Use of Photo-identification and Other Techniques to Estimate Population Parameters.

[61] Davis CC (1948). *Gymnodiniun brevis* sp. Nov., a cause of discolored water and animal mortality in the Gulf of Mexico.. Bot Gaz.

[62] Baden DG, Mende TJ (1982). Toxicity of two toxins from the Florida red tide dinoflagellate, *Ptychodiscus brevis*.. Toxicon.

[63] Odum WE, Steele JH (1970). Utilization of the direct grazing and plant detritus food chains by the striped mullet *Mugil cephalus*..

[64] Moyle PB, Cech JJ (1988). An introduction to ichthyology. 2nd Edition.

[65] Motta P, Clifton KB, Hernandez P, Eggold BT, Giordano SD (1995). Feeding relationships among nine species of seagrass fishes of Tampa Bay, Florida.. Bull Mar Sci.

[66] Woofter RT, Brendtro K, Ramsdell JS (2005). Uptake and elimination of brevetoxin in blood of striped mullet (*Mugil cephalus*) after aqueous exposure to *Karenia brevis*.. Environ Health Persp.

[67] Hinton M, Ramsdell JS (2008). Brevetoxin in two planktivorous fishes after exposure to *Karenia brevis*: implications for food-web transfer to bottlenose dolphins.. Mar Ecol Prog Ser.

[68] Virnstein R, Mikkelsen PS, Kalani DC, Capone M (1983). Seagrass beds versus sand bottoms: The trophic importance of their associated benthic invertebrates.. Florida Sci.

[69] Dawes CJ, Durako M, Phillips K, Lewis R (1987). The dynamic seagrasses of the Gulf of Mexico and Florida coasts.. Proceedings of the Symposium on Subtropical—Tropical Seagrasses of the Southeastern United States.

[70] Sekula-Wood E, Schnetzer A, Benitez-Nelson CR, Anderson C, Berelson WM (2009). Rapid downward transport of the neurotoxin domoic acid in coastal waters.. Nature Geosci.

[71] Mendoza WG, Mead RN, Brand L, Shea D (2008). Determination of brevetoxin in recent marine sediments.. Chemosphere.

[72] Nelson JS (1994). Fishes of the World. 3rd Edition.

[73] Darcy G (1985).

[74] Suzuki C, Hierlihy S (1993). The renal clearance of domoic acid in the rat.. Food Chem Toxicol.

[75] Cattet M, Geraci JR (1993). Distribution and elimination of ingested brevetoxin (PbTx-3) in rats.. Toxicon.

[76] Kvitek RG, Goldberg JD, Smith GJ, Doucette GJ, Silver MW (2008). Domoic acid contamination within eight representative species from the benthic food web of Monterey Bay, California, USA.. MEPS.

[77] Lefebvre KA, Bargu S, Kieckhefer T, Silver MW (2002). From sanddabs to blue whales: the pervasiveness of domoic acid.. Toxicon.

[78] Vigilant VL, Silver MW (2007). Domoic acid in benthic flatfish on the continental shelf of Monterey Bay, California, USA.. Mar Biol.

[79] Burns JM, Ferry JL (2007). Adsorption of domoic acid to marine sediments and clays.. J Envrion Monit.

[80] Gulland FMD, Haulena M, Fauquier D, Langlois G, Lander ME (2002). Domoic acid toxicity in Californian sea lions (*Zalophus californianus*): Clinical signs, treatment and survival.. Veterinary Record.

[81] Levin M, Joshi D, Draghi A, Gulland FM, Jessup D (2010). Immunomodulatory effects upon *in vitro* exposure of California sea lion and southern sea otter peripheral blood leukocytes to domoic acid.. J Wildl Dis.

[82] Levin M, Leibrecht H, Ryan JC, van Dolah FM, De Guise S (2008). Immunomodulatory effects of domoic acid differ between *in vivo* and *in vitro* exposure in mice.. Mar Drugs.

[83] Lefebvre KA, Tilton SC, Bammler TK, Beyer RP, Srinouanprachan S (2009). Gene expression profiles in zebrafish brain after acute exposure to domoic acid at symptomatic and asymptomatic doses.. Toxicol Sci.

[84] Walsh CJ, Leggett SR, Strohbehn K, Pierce RH, Sleasman JW (2008). Effects of *in vitro* brevetoxin exposure on apoptosis and cellular metabolism in a leukemic T cell line (Jurkat).. Mar Drugs.

[85] Murrell R, Gibson J (2009). Brevetoxins 2, 3, 6, and 9 show variability in potency and cause significant induction of DNA damage and apoptosis in Jurkat E6-1 cells.. Arch Toxicol.

[86] Fire SE, Wang Z, Byrd M, Whitehead HR, Paternoster J (in press). Co-occurrence of multiple classes of harmful algal toxins in bottlenose dolphins (*Tursiops truncatus*) stranding during an unusual mortality event in Texas; USA.. Harmful Algae.

[87] Leandro L, Rolland R, Roth PB, Lundholm N, Wang Z (2010). Exposure of the North Atlantic right whale *Eubalaena glacialis* to the marine algal biotoxin, domoic acid.. MEPS.

[88] Hallegraeff GM (1993). A review of harmful algal blooms and their apparent global increase.. Phycologia.

[89] Anderson DM, Glibert PM, Burkholder JM (2002). Harmful algal blooms and eutrophication: Nutrient sources, composition, and consequences.. Estuaries.

[90] Paerl HW, Huisman J (2009). Climate change: a catalyst for global expansion of harmful cyanobacterial blooms.. Environ Microbiol Rep.

