# Supplementary material for: Concurrent Exposure of Bottlenose Dolphins (Tursiops truncatus) to Multiple Algal Toxins in Sarasota Bay, Florida, USA
Source: PLoS One. 2011 Mar 10;6(3):e17394. doi: 10.1371/journal.pone.0017394 (PMC3053359; doi:10.1371/journal.pone.0017394)
Supplement: Table S1 — Concentration of brevetoxin (ng/mL or ng/g) in various animal samples. (PDF) [file pone.0017394.s001.pdf]

Table S1_Concentration of brevetoxin (ng/mL or ng/g) in various animal samples

	June-00		February-04		June-04		February-05		June-05			
	Urine RIA	Serum ELISA	Blood ELISA	Urine LCMS	Urine RIA	Blood ELISA	Urine RIA	Blood ELISA	Urine RIA	Blood ELISA	Serum ELISA	Blood ELISA
FB10												<dl
FB100												<dl
FB101		<dl										<dl
FB109		<dl										
FB11		<dl	<dl									
FB113												<dl
FB114						<dl						
FB117	<dl	<dl										
FB118			0.30				7.10	<dl				
FB119		<dl										
FB123												
FB125		<dl	<dl									
FB127		<dl										
FB13		<dl										
FB133												<dl
FB135							<dl	<dl				
FB137												<dl
FB138					<dl	<dl						
FB14		<dl										
FB141												
FB146		<dl								<dl		
FB148		<dl			<dl	<dl						
FB15		<dl										
FB151												<dl
FB155		<dl								<dl		
FB157												<dl
FB159						<dl						<dl
FB164												<dl
FB173			1.30									
FB174		<dl										
FB175			0.90									
FB177			1.20									
FB178		<dl	<dl									
FB179			0.60	0.53								<dl
FB181					<dl	<dl						
FB182										<dl		
FB185					<dl	<dl						
FB187								<dl				
FB188			0.60		<dl	<dl						
FB189								<dl				
FB193										<dl	<dl	
FB195										<dl	<dl	
FB196		<dl			<dl	<dl						
FB197												
FB198		<dl										
FB199											<dl	
FB20						<dl						<dl
FB203												
FB205												
FB207												
FB209												
FB213												
FB215												
FB217												
FB218							2.10	<dl				
FB219												
FB220							2.90	<dl				
FB224			<dl									<dl
FB226			<dl									
FB228			0.90									
FB230					<dl	<dl						
FB232								<dl				
FB234										<dl	<dl	
FB236										<dl	<dl	
FB238											<dl	
FB240												<dl
FB242												<dl
FB246												<dl
FB25		<dl										
FB250												
FB252												
FB254												
FB256												
FB26		<dl										
FB27			0.70									
FB28		<dl										
FB32		<dl										
FB33		<dl	<dl									<dl
FB36												<dl
FB43		<dl										
FB48		<dl										
FB54												<dl
FB55												
FB58		<dl										
FB65			<dl									
FB7			<dl									
FB75							6.70	<dl				
FB9			1.60									
FB90												<dl
FB92					<dl	<dl						
FB99					<dl	<dl						

Note: '<dl' denotes below limit of detection

June-06			May-08						May-09						
Urine RIA	Feces RBA		Blood RIA/ELIS	Gastric RIA/ELIS	Serum RIA/ELIS	Urine LC/MS	Feces RIA/ELISA		Urine ELISA	Blood ELISA	serum ELISA	feces ELISA	gastric fluid ELISA	milk ELISA	feces (LCMS)
2.60															
3.40															
			<dl	6.70	<dl	<dl	13.60								
0.80	<dl														
									<dl		<dl	32	<dl	<dl	7.9
2.00	<dl		<dl	5.20	<dl	<dl									
									<dl	<dl	<dl	3	<dl		<dl
									<dl	<dl	<dl	<dl	<dl	<dl	
1.60									<dl	<dl	<dl	<dl	<dl		
			<dl	6.50	<dl	<dl	30.00								
26.30	<dl														
32.50															
			<dl		<dl										
			<dl	4.60	<dl	<dl	12.30								
<dl	<dl														
			<dl	4.30	<dl	<dl	2.30								
			<dl	3.70	<dl	<dl									
									<dl		<dl		<dl		
63.40															
			<dl	5.60	<dl	<dl									
			<dl	4.10	<dl	<dl									
			<dl	5.40	<dl	<dl									
			<dl	4.50	<dl	<dl									
									<dl	<dl	<dl		<dl		
										<dl	<dl				
										<dl	<dl				
									<dl	<dl	<dl	5	<dl		<dl
			<dl	<dl	<dl	<dl	13.60								
1.00	109.00														
			<dl	10.50	<dl	<dl	3.50								
									<dl	<dl	<dl	<dl	<dl		
									<dl		<dl	9	<dl		<dl
									<dl		<dl				
89.70															
1.60															
									<dl	<dl	<dl	3	<dl	<dl	<dl
2.40	<dl														
									<dl	<dl	<dl	9	<dl		<dl
