# Supplementary material for: Concurrent Exposure of Bottlenose Dolphins (Tursiops truncatus) to Multiple Algal Toxins in Sarasota Bay, Florida, USA
Source: PLoS One. 2011 Mar 10;6(3):e17394. doi: 10.1371/journal.pone.0017394 (PMC3053359; doi:10.1371/journal.pone.0017394)
Supplement: Table S2 — Concentration of domoic acid (ng/mL or ng/g) in various animal samples. (PDF) [file pone.0017394.s002.pdf]

Table S2_Concentration of domoic acid (ng/mL or ng/g) in various animal samples

	June-00	February-04	June-04		February-05	June-05	
	Urine (mean ELISA/LCMS)	Urine LC/MS	Urine (mean ELI	Blood ELISA	Urine LC/MS	Urine (mean ELI	Blood ELISA
FB10							
FB100							
FB101	<dl						
FB109							
FB11	2.66						
FB113							
FB114				<dl			
FB117	<dl						
FB118							
FB119	6.62						
FB123							
FB125							
FB127							
FB13	9.54						
FB133							
FB135					<dl		
FB137							
FB138			<dl	<dl			
FB14	5.32						
FB141							
FB146	3.10						<dl
FB148			<dl	<dl			
FB15							
FB151							
FB155	1.40					1.32	<dl
FB157							
FB159			6.10	<dl			
FB164							
FB173							
FB174	4.45						
FB175							
FB177							
FB178	<dl						
FB179							
FB181			10.47	<dl			
FB182						16.74	<dl
FB185			5.94	<dl			
FB187							
FB188			1.97	<dl			
FB189							
FB193							
FB195						<dl	
FB196			5.06	<dl			
FB197							<dl
FB198							
FB199						6.03	
FB20			21.80	<dl			
FB203							
FB205							
FB207							
FB209							
FB213							
FB215							
FB217							
FB218					<dl		
FB219							
FB220					<dl		
FB224							
FB226							
FB228							
FB230			1.98	<dl			
FB232							
FB234						7.98	
FB236						9.18	
FB238							
FB240							
FB242							
FB246							
FB25							
FB250							
FB252							
FB254							
FB256							
FB26	3.23						
FB27							
FB28	1.21						
FB32	1.52						
FB33	3.60						
FB36							
FB43	<dl						
FB48	9.50						
FB54							
FB55							
FB58	12.29						
FB65							
FB7							
FB75					<dl		
FB9							
FB90							
FB92			1.04	<dl			
FB99			8.5	<dl			

Note: '<dl' denotes below limit of detection and 'trace' denotes greater than limit of detection but less than limit of quantification

June-06	May-08					May-09					
Urine LC/MS	Blood ELISA	Gastric LC/MS	Serum ELISA	Urine LC/MS	Fecal LC/MS	Urine LC/MS	Blood LC/MS	serum LC/MS	feces LC/MS	astric fluid LC/M	milk LC/MS
<dl											
<dl											
	<dl	<dl	<dl	1.00	10.30						
<dl											
						1.4		<dl	15	<dl	<dl
<dl	<dl	<dl	<dl	<dl							
						3.6	<dl	<dl	11.5	<dl	
						<dl	<dl	<dl	6.5	<dl	<dl
<dl						1.6	<dl	<dl	37	<dl	
	<dl	<dl	<dl	1.60	9.40						
<dl											
<dl											
<dl											
	<dl		<dl								
	<dl	<dl	<dl	<dl	<dl						
<dl											
	<dl	<dl	<dl	<dl	<dl						
	<dl	<dl	<dl	<dl							
						<dl		<dl		<dl	
<dl											
	<dl	<dl	<dl	1.80							
	<dl	<dl	<dl	<dl							
	<dl	13.80	<dl	4.90							
	<dl	<dl	<dl	6.10							
						<dl	<dl	<dl		<dl	
							<dl	<dl			
							<dl	<dl			
						4.6	<dl	<dl	36	2.2	
	<dl	<dl	<dl	17.60	41.50						
<dl											
<dl											
	<dl	<dl	<dl	4.40	8.50						
						trace	<dl	<dl	trace	<dl	
						2.4		<dl	trace	<dl	
						trace		<dl			
<dl											
<dl											
						trace	<dl	<dl	4	<dl	<dl
<dl											
						<dl	<dl	<dl	4	<dl	
